# Mycobiome Dysbiosis in Women with Intrauterine Adhesions

**DOI:** 10.1128/spectrum.01324-22

**Published:** 2022-06-22

**Authors:** Ning-Ning Liu, Xingping Zhao, Jing-Cong Tan, Sheng Liu, Bo-Wen Li, Wan-Xing Xu, Lin Peng, Pan Gu, Waixing Li, Rebecca Shapiro, Xiaoqi Zheng, Wenjing Zhao, Yi-Guo Jiang, Dan Chen, Dabao Xu, Hui Wang

**Affiliations:** a State Key Laboratory of Oncogenes and Related Genes, Center for Single-Cell Omics, School of Public Health, Shanghai Jiao Tong University School of Medicine, Shanghai, China; b Department of Gynecology, Third Xiangya Hospital of Central South University, Changsha, China; c School of Medicine, Shenzhen Campus of Sun Yat-sen University, Sun Yat-sen University, Shenzhen, China; d National Engineering and Research Center of Human Stem Cell, Guangxiu Hospital Hunan Normal University, Changsha, Hunan, China; e Department of Molecular and Cellular Biology, University of Guelph, Guelph, Ontario, Canada; f Department of Mathematics, Shanghai Normal University, Shanghai, China; g The State Key Laboratory of Respiratory Disease, Guangzhou Medical University, Guangzhou, China; h The Third Hospital Affiliated to the Chinese University of Hong Kong Shenzhen, Shenzhen, China; Shenzhen Bay Laboratory

**Keywords:** intrauterine adhesions, reproductive tract, mycobiome, dysbiosis, fungal-bacterial correlation

## Abstract

The vaginal microbiota dysbiosis is closely associated with the development of reproductive diseases. However, the contribution of mycobiome to intrauterine adhesion (IUA) disease remains unknown. Harnessing 16S and ITS2 rDNA sequencing analysis, we investigate both bacterial and fungal microbiota compositions across 174 samples taken from both cervical canal (CC) and middle vagina (MV) sites of IUA patients. Overall, there is no significant difference in microbial diversity between healthy subjects (HS) and IUA patients. However, we observe the IUA-specific bacterial alterations such as increased *Dialister* and decreased *Bifidobacterium* and enriched fungal genera like increased *Filobasidium* and *Exophiala*. Moreover, site-specific fungal-bacterial correlation networks are discovered in both CC and MV samples of IUA patients. Mechanistic investigation shows that Candida parapsilosis, other than Candida albicans and *Candida maltosa*, prevents the exacerbation of inflammatory activities and fibrosis, and modulates bacterial microbiota during IUA progression in a rat model of IUA. Our study thus highlights the importance of mycobiota in IUA progression, which may facilitate the development of therapeutic target for IUA prevention.

**IMPORTANCE** Intrauterine adhesion (IUA) often leads to hypomenorrhea, amenorrhea, repeat miscarriages, and infertility. It has been prevalent over the last few decades in up to 13% of women who experience pregnancy termination during the first trimester, and 30% of women undergo dilation and curettage after a late, spontaneous abortion. However, the pathogenesis of IUA remains unclear. Despite reports of microbiota dysbiosis during IUA progression, there is little information on the effect of fungal microbiota on the development of IUA. This study not only enhances our understanding of the mycobiome in IUA patients but also provides potential intervention strategies for prevention of IUA by targeting mycobiome.

## INTRODUCTION

As one of the most common reproductive diseases in women, the prevalence of intrauterine adhesions (IUA) has increased widely over the past decade, leading to an increased use of intrauterine surgeries including hysteromyomectomy, dilation, and curettage. The most challenging clinical outcomes accompanied with IUA are usually infertility and pregnancy loss (1–5). The main cause of IUA is the severe damage to endometrial integrity, leading to fusion of surfaces of opposing uterine walls (1, 5, 6). To maintain endometrial integrity, there exists three key steps including limiting inflammation, cyclic activation of stem cells, and scar-free repair of the endometrium (7).

Many agents have now been applied to prevent IUA development, such as the absorbable barriers, physical barriers, hormone, cell, or bioagents, etc. (1, 8–17). In spite of advanced technologies, treating IUA is further complicated due to the frequent recurrence of adhesion and risk factors such as delivery, infection, obesity, myomectomy, etc. Thus, strategies attempting to minimize the risk and alleviate its severity are urgently needed.

The pathogenesis of IUA is still under investigation, including the fibrosis hyperplasia theory, abnormal differentiation of stem cells, fibrosis, and alterations in the uterine microenvironment (3, 18). The potential contribution of microbiota inhabited along the female reproductive tract (FRT) to female reproductive health and onset of diseases such as endometrial cancer, endometrial polyps, infertility, and preterm birth are reported (19–22). Specifically, bacterial microbiota and the pathogenic bacterial infection were correlated with the etiology of genital tract and progression of subsequent preterm delivery and late miscarriage (23–27) by regulating the immune responses during pregnancies (22). For instance, Mycobacterium tuberculosis within the genital tract usually can result in severe IUA (18). However, the causal relationship between microbiota and IUA progression remains at its infancy stage. A greater understanding of this pathophysiology of IUA might lead to the development of IUA prevention and treatment strategies.

In this study, we collected 174 samples from 43 IUA patients and 44 healthy subjects (HS) to analyze both fungal and bacterial microbiota present in two different locations of FRT using both 16S and ITS2 rDNA sequencing. Furthermore, we highlighted the important role of the interkingdom microbial correlations in IUA patients. Mechanistically, we elucidated the potential functional role of mycobiome in IUA progression using an IUA rat model.

## RESULTS

### Characteristics of the study population.

**FIG 1:**
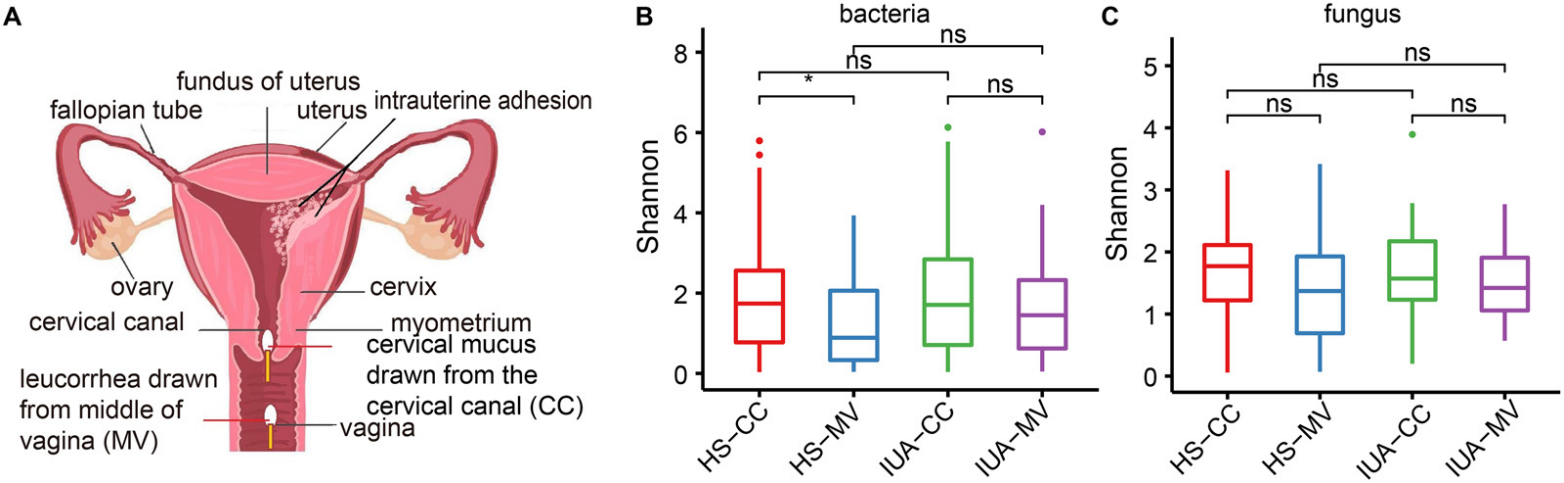
Altered diversity of both bacterial and fungal microbiota. (A) Diagram of the female reproductive tract showing intrauterine adhesions and sampling sites within the cervical canal (CC) and middle vagina (MV) sites. Density plots showing the α-diversity of bacteria (B) and fungi (C), presented as estimated by the Shannon index. HS, healthy subjects; IUA, intrauterine adhesion. *, *P < *0.05; **, *P < *0.01; ns, not significant. *P* values are determined by Kruskal-Wallis test.

In total, 43 IUA patients and 44 healthy subjects (HS) were recruited in this study. All participants were fully informed of the study protocol. The clinical information of the participants including age, height, weight, and body mass index (BMI) are shown in Table 1. The pH value of the IUA patients are provided in Table S1 in supplemental material. No statistical significances of these characteristics were found between IUA and HS. To investigate the vaginal and cervical microbial community structure, a total of 174 samples from both cervical canal (CC) and middle vagina (MV) locations were collected for 16S and ITS2 rDNA sequencing (Fig. 1A; Table S2).

**TABLE 1:** Demographic and clinical characteristics of the study participants^*a*^

Category	HS (*n* = 44)	IUA (*n* = 43)	Odds ratio (95% CI)	*P* value
Age (yr)	31.25 ± 4.44	32.40 ± 4.33	0.667 (0.274–1.677)	0.227
Height (m)	1.59 ± 0.05	1.60 ± 0.04	0.714 (0.296–1.722)	0.192
Weight (kg)	54.27 ± 6.84	56.31 ± 7.14	0.601 (0.256–1.409)	0.177
BMI (kg/m^2^)	21.57 ± 2.43	22.05 ± 2.70	0.760 (0.288–2.005)	0.377

a Values are means ± SD. *P* values were determined by Mann-Whitney test. UA, intrauterine adhesions; HS, healthy subjects; BMI, body mass index; CI, confidence interval.

### Dysbiosis of bacterial and fungal microbiota in IUA patients.

We first compared the α-diversity of bacterial microbiota in MV and CC locations of both HS and IUA groups. In general, we observed no significant difference of α-diversity of both fungal and bacterial communities as indicated by Shannon index in either CC or MV locations between HS and IUA (Fig. 1B and C; Table S3). However, we found a lower α-diversity of bacterial microbiota in HS-MV than HS-CC but not in the case of fungal microbiota. As for β-diversity, a clearly different signature between bacterial and fungal microbiome was found. The samples for bacterial microbiota appeared to be subdivided into two clusters, with the larger cluster indicating a diversified gradient of bacterial communities with less overlap. However, there was no significant difference between MV and CC locations of IUA patients and HS (Fig. S1A). The mycobiome were subdivided into three branches, with one disproportionately larger than the other two, indicating similar mycobiota in most of the samples. However, there were also no obvious differences between these four groups (Fig. S1B).

**FIG 2:**
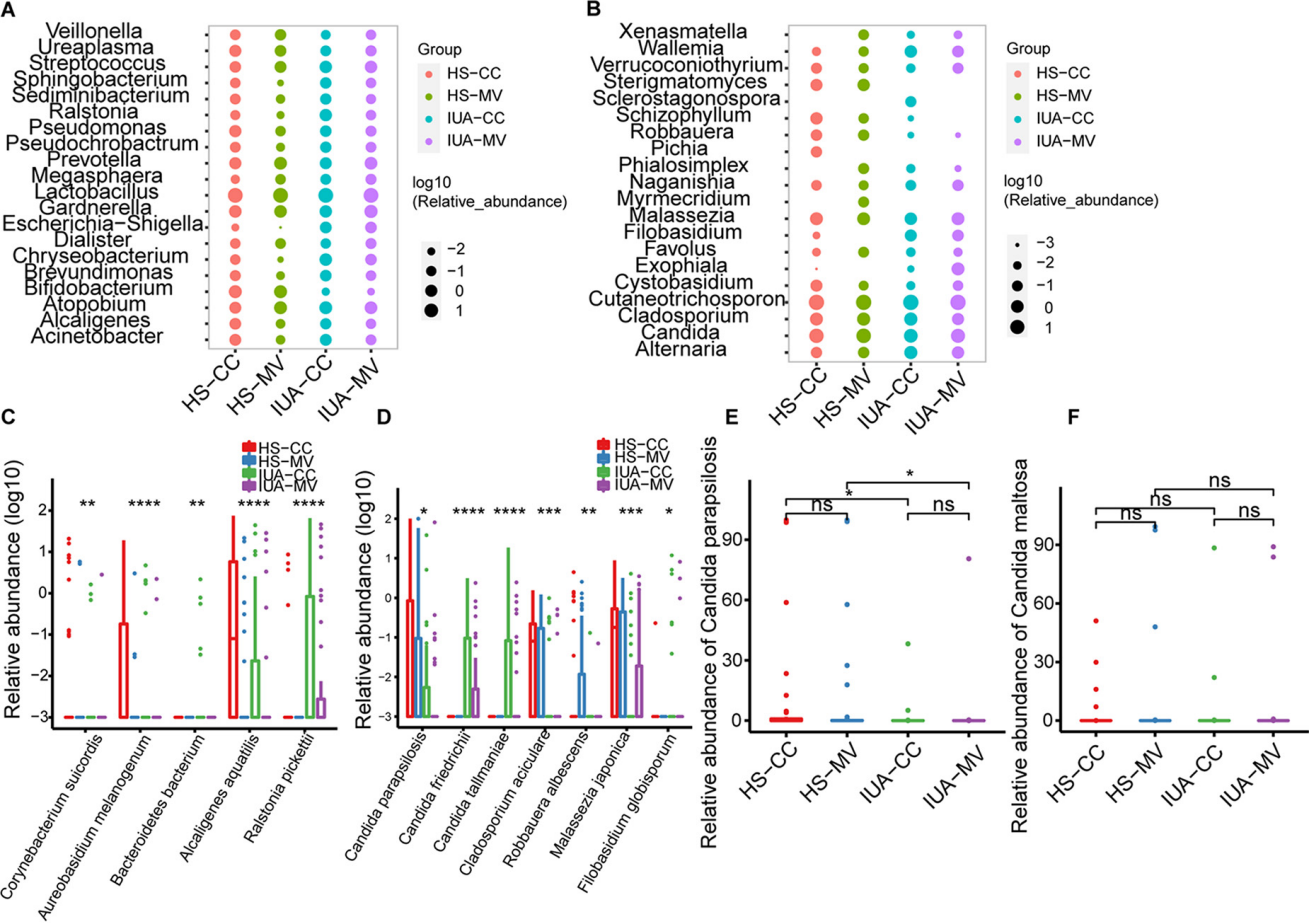
Global composition of bacterial microbiota at the genus (A) and species (C) levels. Global composition of fungal microbiota at the genus (B) and species (D) levels. Relative abundance of Candida parapsilosis (E) and *Candida maltosa* (F) in the lower female reproductive tract (CC and MV) of IUA patients and HS. Average relative abundance is based on circle size. For A to E, IUA CC group, *n* = 43; IUA MV group, *n* = 43; HS CC group, *n* = 44; and HS MV group, *n* = 44. *, *P* < 0.05; **, *P* < 0.01; ***, *P* < 0.001; ****, *P* < 0.0001; ns, not significant. *P* values are determined by Kruskal-Wallis test.

We then examined the microbial composition at both phylum and genus levels. For the bacterial microbiota, *Lactobacillus* was the most abundant genera across all sample locations and groups (Fig. 2A; Fig. S1C) and appeared to be less abundant in the CC site compared to MV within healthy control (Fig. S2A). One striking feature of IUA samples was less *Bafidobacterium* and more *Chryseobacterium* in both CC and MV. Consistent with previous study (28), the relative abundance of *Gardnerella* was higher in IUA patients (Fig. 2A). However, the relative abundance of this genus was still much lower than *Lactobacillus* (Fig. 2A). After assessing the fungal composition across different samples, species from the phylum Basidiomycota greatly predominated the locations of CC and MV (Fig. 2B; Fig. S1D). The genus *Cutaneotrichosporon* was equally distributed across all sampling locations and groups (Fig. 2B). Members of the phylum Ascomycota were the second-most abundant, mostly enriched in the MV than CC. The predominant ascomycete genus was *Candida*, with no significant difference across different groups (Fig. 2B; Fig. S2B). Notably, the relative abundance of species like *Corynebacterium suicordi*, *Aureobasidium melanogenum*, and *Bacteroidetes bacterium* was significantly different among four groups (Fig. 2C). The most abundant *Candida* species was C. parapsilosis, which was more prevalent in HS than in IUA patients, while the relative abundance of *C. maltosa* remained unaltered in IUA patients compared with that in HS (Fig. 2D to F). The typically enriched genera such as *Robbauera* was significantly decreased in HS (Fig. 2B and D). Thus, besides bacterial dysbiosis, our study also uncovered the concomitant differences in fungal microbiota.

Interestingly, we also observed a potential role of vaginal pH in the alteration of IUA microbiota. We have divided the IUA patients into categaries of low pH and high pH based on the healthy reference of vaginal pH (⩽4.6). Then, we compared their microbiota diversity and composition with each other. Although there is no statistically significant difference of both α- and β- diversity for either bacterial or fungal microbiota in the IUA patients with high vaginal pH compared with that of low pH group (Fig. S1E and F), we found an alteration of both bacterial and fungal compositions. For the bacterial microbiota, more *Veillonella* and *Sphingobacterium* and less *Megasphaera* and *Dialister* were observed in the high-pH group than that in the low-pH group (Fig. S1G). For the mycobiome, among the most dominant genera were *Wallemia*, *Sporobolomyces*, *Acremonium*, and *Exophiala* in the high-pH group while *Naganishia*, *Cystobasidium*, and *Cystofilobasidium* were increased in the low-pH group (Fig. S1G). These alterations suggest the potential effect of vaginal pH on microbiota dysbiosis.

Furthermore, our results indicated that the DNA concentration of the negative controls were too low to be used for library construction, excluding the possible contamination during sample collection and DNA extraction.

### Site-specific fungal-bacterial associations in IUA patients.

**FIG 3:**
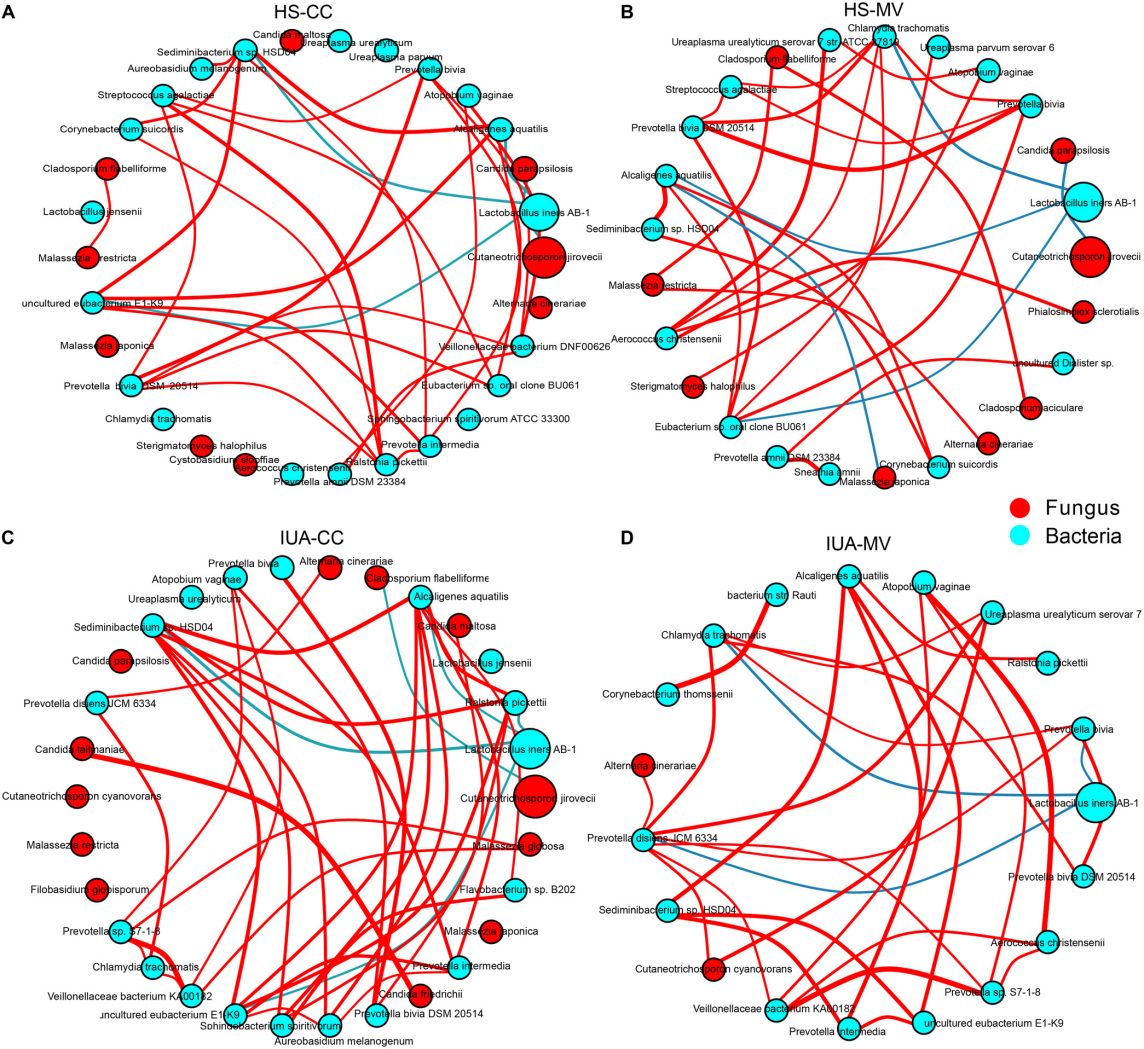
Imbalanced fungal-bacterial correlation networks in both HS (A and B) and IUA patients (C and D) by CytoSCAPE. Each node represents a bacterial or fungal phylum. Node size represents the number of direct edges it has. Edge color indicates the magnitude of the distance correlation (red indicates positive correlation and green indicates negative correlation as determined by the Spearman test). Only the top 30 species in relative abundance with significant correlations (absolute value of correlation coefficient >0.4 and Benjamini-Hochberg adjusted *P* value < 0.05) are displayed.

Fungal-bacterial interactions are reported to be critical determinants in human health and diseases (29–32). To obtain a global view of the fungal-bacterial associations in the genital tract, we built the inter- and intrakingdom correlation network based on the abundance of genera using Cytoscape. As shown in Fig. 3, fungal species from both fungal phyla, namely, Ascomycota and Basidiomycota, established more correlations with bacteria, especially the Proteobacteria in the CC site, of which there were more and stronger positive correlations with basidiomycete. In contrast, Lactobacillus iners *AB-1* was notably negatively associated with uncultured eubacterium E1-K9 in CC samples, suggesting the potential stable correlation (Benjamini-Hochberg [BH] adjusted *P < *0.05; Table S4 and S5). In addition, there was a significant negative correlation between Candida parapsilosis and *Cutaneotrichosporon jirovecii* in HS group (*r* = −0.47 and *r* = −0.49 for CC and MV, respectively); this correlation was not found in IUA patients. Furthermore, the relative abundance of *Candida maltose* was negatively associated with Prevotella bivia in CC samples of IUA patients (*r* = −0.48).

### Functional signatures of IUA microbiota.

Due to the microbial heterogeneity between IUA patients and HS, we explored the functional alterations at KEGG orthologue (KO) genes and pathway level at both CC and MV sites, respectively. We identified 395 differential KO genes with increased abundance in the MV of IUA patients compared to healthy control (Table S6). At pathway level, there were 20 identified pathways enriched in the MV of IUA patients (Table S7), including glycolysis III, peptidoglycan biosynthesis I, and CDP-diacylglycerol biosynthesis. In contrast, no differential KO genes or pathways were identified in CC. These results indicate a potential site-dependent effect of microbiota on IUA progression.

### Exacerbation of inflammatory activities and fibrosis in IUA patients.

**FIG 4:**
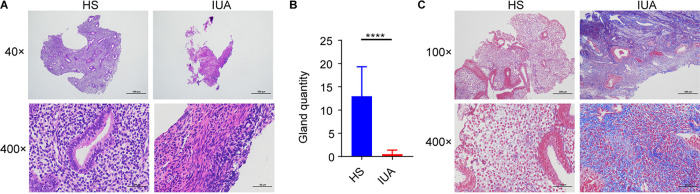
H&E and Masson staining of endometrial tissue from IUA patients. (A) hematoxylin and eosin (H&E) staining of endometrial tissues in HS (left) and IUA patients (right) shown at ×40 and ×400 magnifications. (B) Bar chart illustrating differences in gland quantity for endometrial tissue from IUA patients (*n* = 10) and HS (*n* = 10). (C) Masson staining of endometrial tissue in HS (left) and IUA patients (right). In A to C: ****, *P < *0.0001. *P* values are determined by two-tailed Student's *t* test (B).

**FIG 5:**
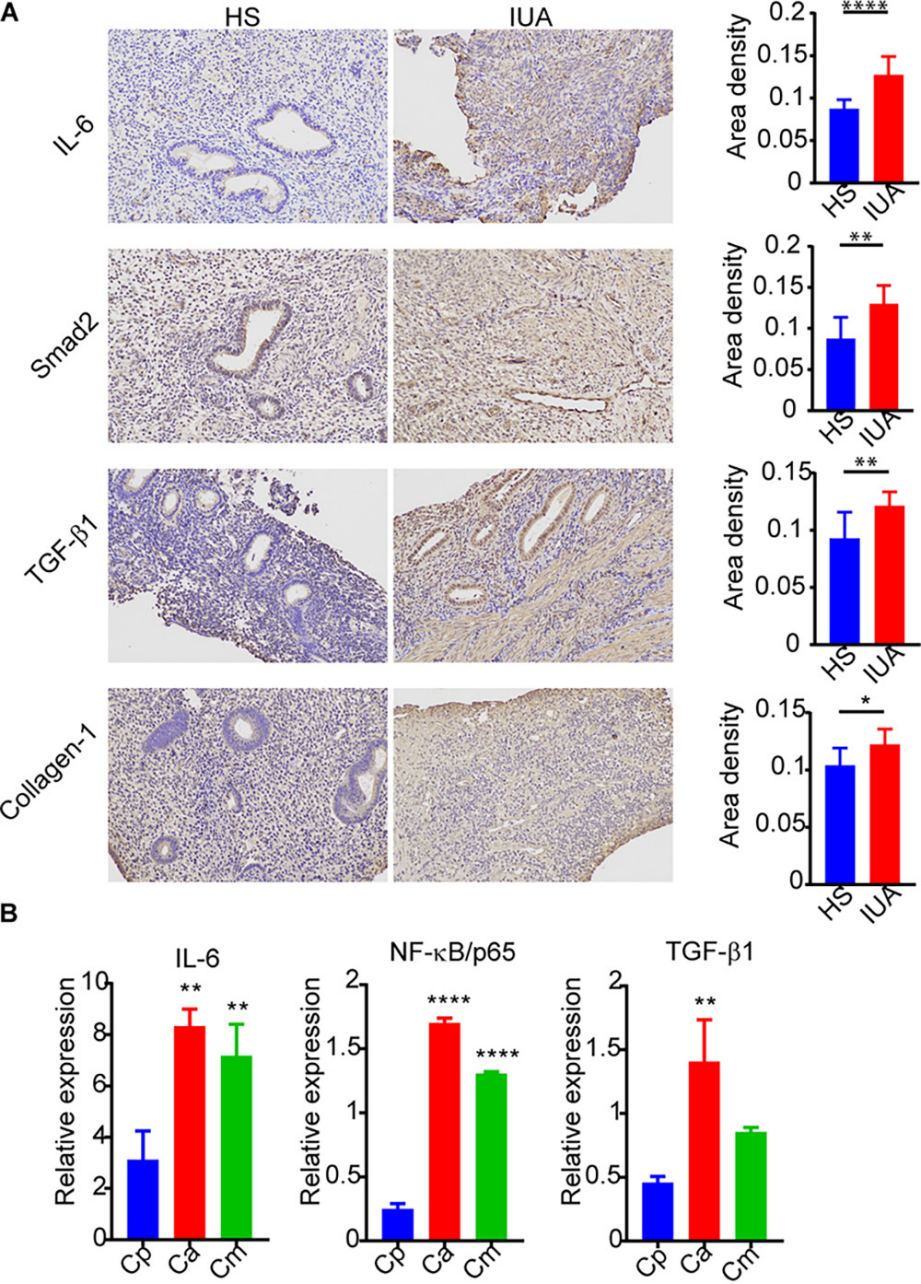
Exacerbated inflammatory activities and fibrosis induced during IUA. (A) Immunohistochemistry assay of endometria tissues from IUA patients and healthy subjects. (B) Bar graphs showing inteluekin-6 (IL-6), NF-κB/p65, and transforming growth factor-β1 (TGF-β1) expression in primary endometrial cells stimulated by Candida albicans (Ca), C. parapsilosis (Cp), and *C. maltose* (Cm) as quantified by quantitative RT-PCR. In A and B: *, *P < *0.05; **, *P < *0.01; ****, *P < *0.0001. The *P* values are determined by two-tailed Student’s *t* test (A) and one-way ANOVA and Dunnett *post hoc* tests (B).

We performed the hematoxylin and eosin (H&E; Fig. 4A and B) and Masson staining to evaluate the inflammatory activities and fibrosis in IUA patients (Fig. 4C). It revealed that there were less endometrial glands and well-arranged connective tissue with more collagen fibers in IUA patients (Fig. 4). Furthermore, our immunohistochemistry (IHC) assay showed increased expression of the collagen-1, Smad2, transforming growth factor-β1 (TGF-β1), and interleukin-6 (IL-6), which are known fibrosis biomarkers (33–37) (Fig. 5A). These findings highlight the inflammatory activities and exacerbated fibrosis associated with IUA progression. Furthermore, compared with *C. maltosa*, C. parapsilosis was decreased in IUA patients (Fig. 2E). Consistently, the expression of IL-6, NF-κB/p65, and TGF-β (Fig. 5B) was significantly decreased following incubation of endometrial cells with C. parapsilosis, but not with C. albicans or *C. maltosa*. These results indicate that C. parapsilosis might be correlated with the inflammatory activities and fibrosis in IUA patients.

### Protective benefits of C. parapsilosis in a rat model of IUA.

**FIG 6:**
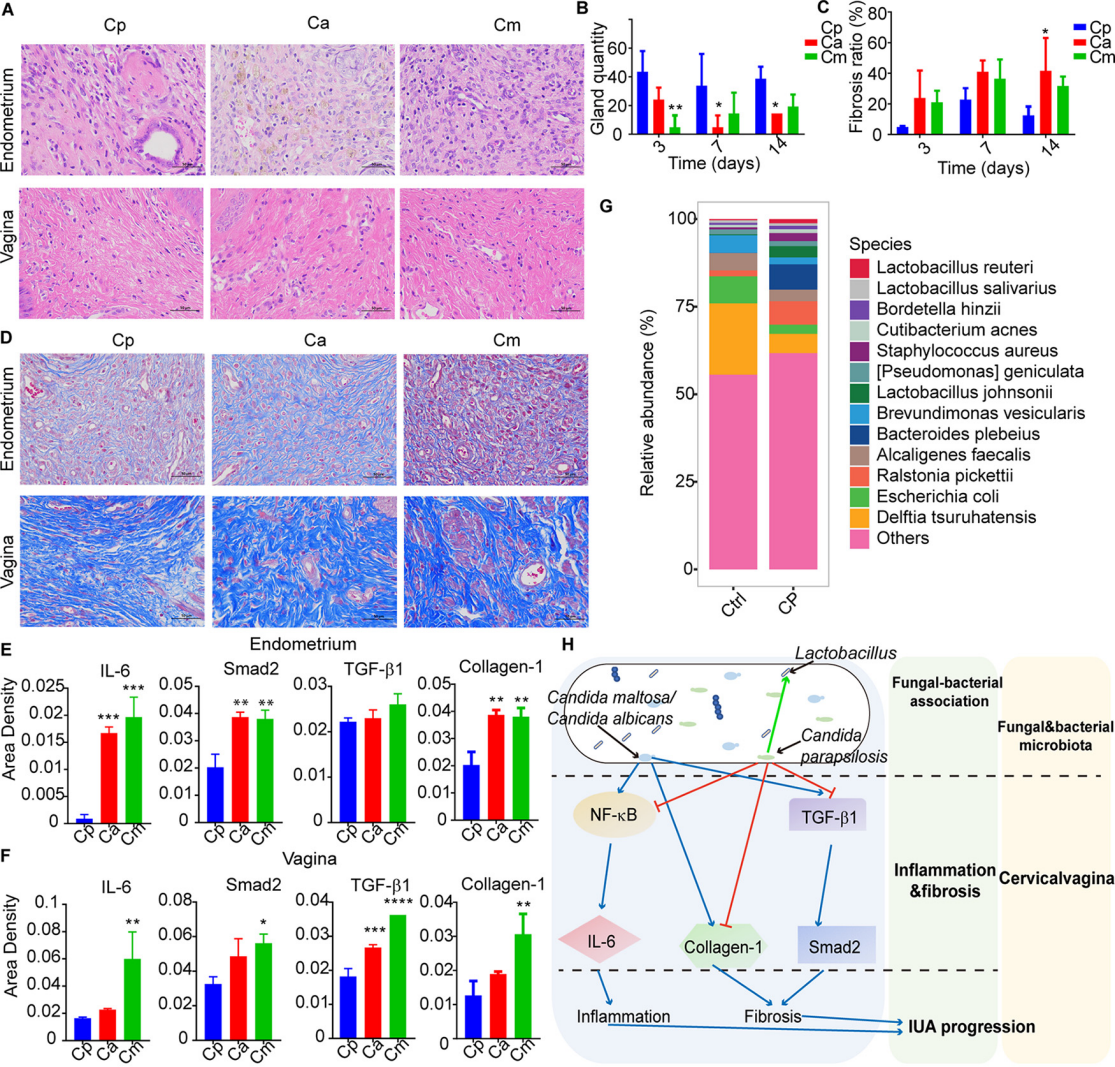
Protective benefits of C. parapsilosis against IUA progression in rat models. (A) H&E staining of rat endometrial and vaginal tissues containing each of three *Candida* species. (B) Bar graphs showing representative gland quantity in endometria of rats from each *Candida* group. (C) Masson staining of rat endometrial and vaginal tissues exposed to each *Candida* species examined. (D) Bar graphs for representative fibrosis area ratios of endometria in rats for each *Candida* group over three time points. (E and F) Bar graphs showing immunohistochemistry results of Smad2, IL-6, and TGF-β1 in endometrial tissue (E) and vaginal tissue (F). (G) Global dysbiosis of bacterial microbiota composition at the species level for both control (left) and Cp (right) groups after 14 days (*n* = 6). (H) Diagram showing how mycobiome dysbiosis, especially from *Candida* spp, contributes to IUA progression through regulation of inflammation and fibrosis, and through change of bacterial microbiota. In all panels: *, *P < *0.05; **, *P < *0.01; ***, *P < *0.001; ****, *P < *0.0001. The *P* values are determined by one-way ANOVA and Dunnett *post hoc* tests (B, D, and E). Cp, Cp-treated group (*n* = 6); Ca, Ca-treated group (*n* = 6); Cm, Cm-treated group (*n* = 6).

To confirm the above findings, we infected the rats with C. parapsilosis and observed less fibrosis and damage to the endometrium compared with C. albicans or *C. maltosa* in the rat model of IUA (Fig. 6A to D). Specifically, there were less epithelial cell exfoliation and larger gland numbers and inflammatory cell infiltration after treatment with C. parapsilosis (Fig. 6A and B). Furthermore, the immunohistochemistry analysis of collagen-1, Smad2, IL-6, and TGF-β1 expression were remarkably decreased in both the endometrial and vaginal samples of C. parapsilosis group (Fig. 6E and F; Fig. S3). To examine the corresponding bacterial dysbiosis, we performed the 16S rDNA sequencing by extracting microbial DNA from rat endometrium and vaginal tissues. The results revealed that the α-diversity in vaginal tissue and the β-diversity of endometrium were significantly higher in the C. parapsilosis group (*P < *0.05) (Fig. S4A and B). The bacterial features associated with C. parapsilosis in endometrium included the widely recognized probiotic bacteria such as *Lactobacillus* (e.g., Lactobacillus johnsonii and Lactobacillus reuteri), for which the abundance was greatly increased (Fig. 6G; Fig. S4C). Proteobacteria was the most abundant phylum in both groups. The relative abundance of Bacteroidota and Firmicutes was increased after stimulation with C. parapsilosis (Fig. S4D) with the exception of genus Delftia tsuruhatensis and Escherichia coli (Fig. 6G). Taken together, our study indicates a protective effect of C. parapsilosis against IUA progression, accompanied with the alteration of bacterial microbiota in the reproductive tract (Fig. 6H).

## DISCUSSION

Disruption of the microbial ecosystem in the female reproductive tract is closely related with development of reproductive diseases (38). During the progression of reproductive diseases, vaginal microbiome is reported to intensively interact with the host immune system (39–43). However, the CC microbiome, especially its fungal microbiota and their associations with bacterial microbiota, has not been previously explored. In this study, we not only characterized the disease-specific cervical-vaginal mycobiome in IUA, but also highlighted the contribution of mycobiome to IUA pathogenesis. Specifically, an unexpected protective benefit of C. parapsilosis against IUA progression was discovered in the rat model of IUA, accompanied with the alteration of bacterial microbiota in the lower reproductive tract.

The CC represents a unique ecological niche, as indicated by the heterogeneity of microbiota between this location and that of the MV. In IUA patients, compared to MV, the CC sites possessed a relatively higher α-diversity of both bacterial and fungal microbiota, although not statistically significant, with stronger fungal-bacterial associations. Furthermore, the relative abundance of microbial community at these two sites was described as inversely proportional. For example, the relative abundance of *Verrucoconiothyrium prosopidis* (fungus) is decreased in the CC but increased in the MV in IUA patients. These results suggest the niche dependent microbiota alteration in both MV and CC.

The causal relationship between microbiota dysbiosis and pathogenesis in IUA patients remains one of the chicken-and-egg problems. The *Lactobacillus* species that dominated the bacterial microbiota of both sampling sites have been shown to protect the inhabited microenvironment from invading pathogens through production of antimicrobial products and blockage of adhesion (44–46). Our finding showed that abundances of *Lactobacillus* was not significantly altered in IUA patients compared to healthy controls for both CC and MV samples, indicating the functional role of other microbes such as fungi (Fig. S2). Further investigations are required to elucidate the functional connections between disturbed microbiome and IUA progression.

Although frequent fungal infections significantly distorted the inherent vaginal micro-ecosystem, most studies tend to neglect the mycobiome due to the difficulty in its low biomass, DNA extraction, and annotation (46). Furthermore, both fungal and bacterial communities are required to maintain balanced microbiota (46, 47). In addition, site-specific interkingdom correlations between fungi and bacteria were observed in the CC site of IUA patients, suggesting complex fungal-bacterial interactions predominating in the cervical-vagina region of IUA patients and the critical role of fungal mycobiota in IUA progression. Combination of bacteria and fungi may yield a favorable niche (48). The mechanisms by which fungi and bacteria regulate each other in these commensal communities are undoubtedly different and may offer novel targets for manipulation of the dysregulated microbiome.

It has been reported that fungi also induce protective benefits in the microbiome (47). As a non-*albicans Candida* species, C. parapsilosis was enriched in the lower reproductive tract (46) and often used as a low pathogenicity control (49). It was unlikely to invade and damage host tissues similar to C. albicans due to its potential to protect the host from infection by C. albicans (50, 51). Moreover, in an animal model of experimental autoimmune encephalomyelitis (EAE), it was reported that C. parapsilosis infection did not exacerbate the severity of EAE and exhibited the lowest fungal burden in the central nervous system (52). Furthermore, the yeast-hyphal morphogenesis is reported to be critical for fungal virulence (53). However, compared to C. albicans, which can dynamically transit between yeast and hyphae morphologies, C. parapsilosis remain in the yeast form when cultured in microglia medium (54). During vulvovaginal candidiasis, C. parapsilosis was grown in the yeast morphology compared to the long hyphae formed by C. albicans, which formed cell aggregates without causing invasion or damage (55). Furthermore, we demonstrated that C. parapsilosis decreased the inflammatory activity by downregulating expression of NF-κB and IL-6 and restrained fibrosis by suppressing expression of the TGF-β1, collagen-1, and Smad2 biomarkers compared to other *Candida* species.

In the rat model of IUA with Candida infection, we used heat-killed fungal cells, which ensured consistency in the physiological state of the cells of the inoculum used in these experiments. First, the live fungal cells will proliferate and transit between yeast and hyphae morphologies in the host niche of reproductive tract, which might cause invasion into the tissue and lead to mucosal fungal infection (56, 57). Second, the use of heat-killed fungal cells will avoid the indirect effect of secreted factors by live fungal cells. Third, fungal cells killed by heating will still maintain the integrity of the fungal cell structure such as cell wall components which can be recognized by several receptors on the host cell surface, including the Toll-like receptor 2. For example, heat-killed fungal particles were reported to induce an inflammatory response in 3T3-L1 adipocytes and the innate immune defense by host cells (58–60).

*Lactobacillus* is often the dominant bacterial genus within the FRT and can promote vaginal health by competitive exclusion and modulate the FRT environment through the production of lactic acid and bacteriocins (44, 61–63). As an opportunistic pathogen causing bacteremia in immunocompromised individuals, Brevundimonas vesicularis has been isolated from cervical specimens, which supports the growth of *Legionella* (64). Delftia tsuruhatensis is another opportunistic and emerging health care-associated pathogen involved in urinary tract infections (65, 66) that has potential to synergistically interact with E. coli, which results in increased antibiotic resistance (65). Our study suggests the potential of C. parapsilosis in modulating bacterial microbiota. Thus, therapeutic manipulation of fungal mycobiota like C. parapsilosis could be exploited as an effective approach for treatment or prevention of IUA progression.

Finally, one potential limitation of this study might be the use of older version of Silva and UNITE databases and 2018 version of QIIME2. The composition of microbiota and interaction network between fungi and bacteria might be altered based on the new versions of database of pipeline tools. This is inevitable as the new pipeline tools have always sprung up like mushrooms. However, the major species of our interest, such as Candida parapsilosis, will not change significantly with different versions of database of pipeline tools. To gain a systematic investigation of the core bacterial or fungal species, metagenomic sequencing with cross-cohort validation will be required to facilitate the accurate identification of IUA biomarkers and interkingdom association network.

In conclusion, we observed IUA-specific signatures of fungal-bacterial interaction networks in IUA patients and discovered C. parapsilosis could contribute a beneficial effect against inflammation and fibrosis during adhesion progression through NF-κB-mediated IL-6 inactivation. Understanding how fungi contributes to the microbiome and host immune systems may lead to novel therapeutic approaches for better clinical management and prevention of IUA.

## MATERIALS AND METHODS

### Patients and public involvement.

Patients were not involved in the design, experimentation, reporting, or dissemination of our research.

### Sample collection.

This study involved samples taken from 43 women, between 20 and 40 years of age and newly diagnosed with IUA during hysteroscopy examination at the Third Xiangya Hospital of Central South University. IUA patients were scored according to the American Fertility Society (AFS) classification system (IUA group) by the surgeon (67). The intrauterine adhesions were scored as follows: 1–4 (mild); 5–8 (moderate); and 9–12 (severe). In addition, 44 women of the same age range, found to be healthy (HS) based on a routine gynecological health examination in the same hospital and during the same period, were recruited as the control group. None of the participants received vaginal medication or cervical treatment or had performed douching within the 7 days prior to sample collection. None of the participants had engaged in sexual activity within at least 2 days prior to sample collection. Furthermore, all women in this study had a history of uterine cavity operation but were not diagnosed as having endocrine or autoimmune disorders, cancer, severe pelvic adhesions, hysteromyoma, endometriosis, adenomyosis, or acute inflammation. The samples were collected by swabs, including leucorrhea, drawn from the MV, and as cervical mucus drawn from the CC in the follicular phase of the menstrual cycle. To examine the potential contamination, we also set blank controls at each step including sample collection and DNA extraction. All swabs were immediately stored at −80°C until microbial DNA extraction.

### DNA preparation.

Microbial DNA was extracted from 186 samples using a Fast DNA SPIN Kit for Soil (cat no. 116560200MP; Biomedicals) according to manufacturer’s instructions (68). During the DNA extraction process, we set environmental controls to discard the potential contamination. The concentration and quality of purified DNA were determined via a spectrophotometer at the wavelength of 230 nm (A_230_) and 260 nm (A_260_; NanoDrop).

### 16S and ITS2 rDNA sequencing and analysis.

Bacterial composition and diversity were determined by 16S rDNA sequencing. The V4 and V5 regions of 16S ribosomal DNA were amplified. ITS2 gene sequencing was used to assess the mycobiome (fungal composition) wherein the gene was amplified described previously (69). The primers used for amplification were included in Table S1. The PCR cycling conditions involved 35 cycles at 93°C for 20 s, 50°C for 30 s, and 72°C for 1 min with purified microbial DNA as a template. The PCR products were then mixed with 108 barcoded primers (Acebiox Primers) and subjected to further cycling conditions of eight cycles at 93°C for 20 s, 60°C for 20 s, and 72°C for 1 min. The final PCR products were then purified with a gel extraction kit (cat. no. CW2302S; CWbio) and quantified using an ultra-micros spectrophotometer (DS-11; DeNovix, USA). All sequencing analyses were performed using a 300-bp paired-end sequencing protocol on an Illumina MiSeq platform (Illumina, San Diego, CA, USA). The raw sequencing data were processed as follows: (i) a PRINSEQ-lite PERL script was used for quality filtering by truncating the bases from the 3′ end that did not show a quality <30 based on the Phred algorithm; (ii) Fast length adjustment of short reads was applied for paired-end read assembly to improve genomic assemblies with a minimum overlap of 30 bases and a 97% overlap identity; and (iii) CutAdapt was used to search for and remove both forward and reverse primer sequences to ensure there were no mismatches. Assembled sequences, for which ideal forward and reverse primers were not found, were eliminated.

The sequences were demultiplexed and filtered through QIIME2 (https://qiime2.org/) and then assigned to operational taxonomic units (OTUs) with a 97% pairwise identity threshold. They were classified taxonomically using the Silva 132 (16S rRNA) database for bacteria and the UNITE ITS database (alpha version 12_11) for fungi. The α–diversity was estimated by QIIME2 (2018.4) for diversity index and the number of observed species. The β-diversity was measured by QIIME2 (2018.4), and a Bray-Curtis distance matrix was used to build principal coordinate analysis plots. Bubble plots of relative taxonomic abundances were generated using the R package “ggplot” to assess both bacterial and fungal microbiota in HS and IUA. LefSe analyses were performed using LefSe version 1.0 and DESeq2 through the R package. The analysis of fungal-bacterial correlations, and generation of the imbalanced trans-kingdom network, were conducted as previously described (70).

### Coincubation of *Candida* species and primary endometrial cells.

Primary endometrial cells were collected from both IUA and HS, cultured in Dulbecco’s Modified Eagle’s Medium/Nutrient Mixture F-12 (DMEM/F12) supplemented with fetal bovine serum (10% vol/vol), and incubated with 5% CO_2_ at 37°C. Primary endometrial cells were stimulated with heat-killed (65°C water bath for 1 h) C. albicans (BWP17 + Clp30, M1477), C. parapsilosis (YCB330), or C. maltosa (CGMCC 2.1974 = ATCC 28140), at a multiplicity of infection (MOI) of 5 for 6 h. The heat-killed *Candida* cells were cultivated on YPD plates for further 72 h to insure that all cells were inactivated. RNA was then extracted for quantitative RT-PCR assay.

### Rat model of IUA with *Candida* infection.

The IUA rat models used in our study were previously established (71). Briefly, a total of twenty-four 13- to 15-day pregnant Sprague-Dawley rats, aged 10 weeks and weighing between 400 and 450 g, were purchased from Hunan SJA Laboratory Animal Co., Ltd. Before surgery, all rat subjects were housed individually, with free access to a standard diet and sterile water, at a temperature of 23 ± 2°C with 12 h of light and 12 h of darkness.

Experimental surgery in pregnant rats was performed by the same person. The details of the procedures are as follows: after anesthesia (intraperitoneal administration of sterile sodium pentobarbital, 30 mg/kg) and disinfection with iodine, a 4-cm longitudinal midline incision of the lower abdomen was made to access the abdominal cavity, and both sides of the uterus were exposed. A 1-cm longitudinal uterine incision was cut close to the proximal end of the uterus, and the bilateral embryos were completely removed. The rat endometrium was scraped with a curette in four different directions (front, back, left, and right). No other procedures were performed until the embryo was completely removed. After suturing the uterine and abdominal wall incisions, 50 μL of fungal cell suspensions (containing 3 × 10^6^ cells that had been heat killed in a 65°C water bath for 1 h) of C. albicans (BWP17 + Clp30, M1477), C. parapsilosis (YCB330), or C. maltosa (CGMCC 2.1974 = ATCC 28140) were injected into the cervical canal by gavage, while the control group was injected with 50 μL of phosphate-buffered saline (PBS). The rats were individually euthanized and dissected on either the 3rd, 7th, or 14th days after their surgery. We then compared tissue morphology, the number of endometrial glands, the degree of endometrial fibroses, and the expression levels of IL-6, TGF-β1, smad2, and collagen-1 in endometrial and vaginal tissues between the experimental group and control group.

### Tissue sample collection.

Endometrial and vaginal tissue samples were collected from the IUA rat models, and endometrial tissue samples were collected from 10 IUA human patients and 10 healthy subjects. Each sample was divided into two parts: one part was kept at room temperature in formalin and the other part was stored at −80°C.

### Histopathological analysis.

Paraffin sections were made for tissue specimens fixed in 4% neutral buffered formalin. These were stained with H&E (cat. no. G1005; Servicebio) and Masson trichrome (cat. no. G1006; Servicebio) according to the protocol provided by the manufacturer and then scanned using a three-dimensional panoramic scanner (3D HISTECH Pannoramic 250; made in Hungary). The number of endometrial glands present in each H&E section of endometrial tissue was counted under a ×40 microscope. Three fields of each Masson section were randomly selected under high magnification (×100), and the image analysis system, Image-Pro Plus 6.0, was used to calculate the area of endometrial stromal fibrosis. The fibrosis ratio was calculated as the area of the endometrial stromal fibrosis in each field divided by the total area of the endometrial interstitium and glands.

### DNA preparation from formalin-fixed, paraffin-embedded tissues and 16S rRNA sequencing.

Microbial DNA was extracted from rat endometrial and vaginal tissues with a QIAamp DNA FFPE Tissue Kit (cat. no. 56404; Qiagen) according to the manufacturer’s instructions. The concentration and quality of purified DNA were determined via a spectrophotometer at the wavelength of 230 nm (A_230_) and 260 nm (A_260_; NanoDrop).

Bacterial composition and diversity were determined by 16S rRNA sequencing. The V4 and V5 region of 16S ribosomal DNA was amplified using Phusion High-Fidelity PCR Master Mix with GC Buffer (cat. no. 0532; NEB) (72). The primers used for amplification are included in Table S1. The final PCR products were then purified using a gel extraction kit (cat. no. 28704; Qiagen). A TruSeq DNA PCR-Free Sample Preparation Kit (cat. no. 20015960; Illumina) was used for library construction.

All the sequencing analyses were performed using a 250-bp paired-end sequencing protocol on a NovaSeq600 machine (Illumina, San Diego, CA, USA). Paired-end reads were assigned to samples based on their unique barcodes and then trimmed by cutting off the barcode and primer sequence, and finally merged using FLASH (V1.2.7, http://ccb.jhu.edu/software/FLASH/). Quality filtering on the raw tags with specific filtering conditions was conducted to obtain the highest quality of clean tags according to the QIIME2-2018.4 quality control process. The tags were compared with the reference database (Silvadatabase, https://www.arb-silva.de/), using the UCHIME algorithm (UCHIME, http://www.drive5.com/usearch/manual/uchime_algo.html) to detect chimera sequences, which were subsequently removed.

Sequence analyses were performed with Uparse software (Uparse V7.0.1001, http://drive5.com/uparse/), and those with ≥ 97% similarity were assigned to the same OTUs. A representative sequence for each OTU was screened for further annotation using the Silva Database (http://www.arb-silva.de/), based on the Mothur algorithm to annotate taxonomic information. In order to study phylogenetic relationships among the different OTUs, and assess the dominant species in different samples, multiple sequence alignments were conducted using MUSCLE software (V3.8.31, http://www.drive5.com/muscle/). OTU abundance information was normalized using a standard of sequence count based on the sample with the least number of sequences. Subsequent analyses of α-diversity and β-diversity were performed using this normalized data. The α-diversity was calculated with QIIME2 and displayed with R software (V3.5.0). The β-diversity on unweighted unifrac was also calculated with QIIME2.

### Immunohistochemistry assay.

The specimen sections were first dewaxed and hydrated. After high-temperature and high-pressure antigen repair, sections were incubated with 3% H_2_O_2_ at room temperature for 25 min to block endogenous peroxidase activity and then 3% BSA at room temperature for 30 min. Antibodies (anti-smad2, cat. no. ab40855, abcam; anti-collagen-1, cat. no. ab34710, abcam; anti-IL-6, cat. no. ab9324, abcam; and anti-TGF beta 1, cat. no. ab92486, abcam) were each added at a dilution of 1:200 and incubated at 4°C overnight. PBS was used as the negative control. The specimens were removed and rewarmed at room temperature for 30 min after being washed with PBS. After incubation with 3,3′-diaminobenzidine at room temperature for 30 s, the specimen sections were counterstained with hematoxylin. Slides were dehydrated with alcohol, cleared with xylene, and sealed with neutral gum. Three fields were randomly selected under a high-powered lens (×200) for each section, and there was no blank area in each field. Images of stained endometrial IHC sections were captured and measured using Image-Pro Plus 6.0 image analysis software. For the integrated optical density (IOD), three to four fields from each section were randomly selected, and their IOD values, as well as the area of their endometrial proteins, were then calculated. Area density (IOD/area) was used to evaluate the expression level.

### Quantitative RT-PCR analysis.

Total RNA was extracted using TRNzol Universal Reagent (cat. no. DP424; Tiangen), and cDNA was reverse transcribed by the PrimeScript RT reagent kit with gDNA Eraser (Perfect Real Time) (cat. no. RR047A; TaKaRa). According to the manufacturer’s instructions, quantitative RT-PCR was performed using TB Green Premix *Ex Taq* II (Tli RNaseH Plus) (cat. no. RR820A; TaKaRa). Expression levels were normalized to those obtained with the control gene β-actin. The primers used for amplification were included in Table S1.

### Wound healing assay.

Primary endometrial cells were seeded in a six-well plate to confluent state, and three scratches were created with a sterile 200-μL pipette tip. The cells were then incubated in FBS-free DMEM/F12 medium, either alone or infected with heat-killed C. parapsilosis at an MOI = 5. Finally, the cells were photographed at 0 h and 24 h with an Olympus IX73 microscope at ×100 magnification, and then analyzed using ImageJ software.

### Correlation between cytokine expression and fungal abundance.

To calculate the correlation between cytokine expression and proportions of fungal species measured by their ITS2 reads at different levels, we performed a linear regression model with Pearson/Spearman correlation coefficients and *P* values for each condition. The coefficient of the cytokine expression term is significant if its regression *P* value (RegPval) is <0.05. Confounding factors such as age, height, weight, BMI, and AFS score may have potential effects on cytokine expression.

### Statistical analysis.

All clinical data are expressed as median ± SD and analyzed using a two-tailed Student's *t* test, a Mann-Whitney *U* test, or a one-way ANOVA with IBM SPSS Statistics 20. The QIIME2 (Quantitative Insights into Microbial Ecology) platform was used to detect and evaluate the data. For α-diversity, the Shannon index was tested by Kruskal-Wallis method to compare differences between groups, while β-diversity was analyzed by principle coordinate analysis using the pseudo-F (permanova) test based on the Bray-Curtis distance. Linear discriminant analysis effect size (LefSe) analysis, including the cladogram, was used to identify microbial biomarkers with statistical significance between groups. DESeq2 (one of the analytical methods recommended by QIIME2) was applied based on the corrected *P* value of <0.01 with fold changes >2 or <0.5. Global fungal-bacterial correlation networks were built with Cytoscape V3.4.0 (https://cytoscape.org/), and Spearman test was used to calculate the correlation of species based on relative abundances, with *P*-value correction (BH method, *P < *0.05).

### Study approval.

For experiments utilizing human swab samples whose donors were identifiable, written informed consent was obtained from study subjects, according to protocols approved by The Institutional Review Board (IRB) of Third Xiangya Hospital, Central South University. The procedure was approved by the IRB of Third Xiangya Hospital, Central South University under the permit number 2018-S001.

All animal experiments were performed in accordance with recommendations in the *Guidance for the Care and Use of Laboratory Animals* of the National Institutes of Health. The Ethics Committee of Experimental Animal Welfare, Central South University reviewed and approved the establishment of the animal model of intrauterine adhesions after surgical abortion and curettage in pregnant rats. All experimental procedures were approved by The Ethics Committee of Experimental Animal Welfare, Central South University under the permit number 2020sydw0713.

### Data and materials availability.

The raw sequencing data sets are publicly available in the NCBI database with BioProject accession numbers PRJNA640979 and PRJNA724684 upon publication. All codes for the linear regression model are available at https://github.com/vivislan/R-for-linear-regression.git. The supplemental materials including the Supplemental Fig. S1–S4 and Tables S1–S7 are all available online along with the manuscript.
