# Supplementary material for: Mycobiome Dysbiosis in Women with Intrauterine Adhesions
Source: Microbiol Spectr. 2022 Jun 22;10(4):e01324-22. doi: 10.1128/spectrum.01324-22 (PMC9431258; doi:10.1128/spectrum.01324-22)
Supplement: Supplemental file 1 — Fig. S1-S4; captions for Tables S1-S7. Download spectrum.01324-22-s0001.pdf, PDF file, 1.1 MB [file spectrum.01324-22-s0001.pdf]

Supplemental Material

Supplemental Tables S1-S7

Table S1. Vaginal pH of the IUA patients.

Table S2. Primers used in this study.

Table S3. The Shannon index of 16S rDNA sequencing samples and ITS2 rDNA sequencing samples.

Table S4. The disease specific fungal-bacterial microbiota correlation at the genus level.

Table S5. The spearman correlation matrix in samples from two sites (CC and MV) of HS and IUA.

Table S6. Differential KO (KEGG Ontology) genes in MV samples between IUA and HS.

Table S7. Differential pathways in MV samples between IUA and HS.

Supplemental Figures S1-S4

Figure S1. Principal coordinates analysis of both bacterial and fungal microbiota. Plots showing the diversity of bacteria (A) and fungi (B) in the lower FRT (CC and MV) of IUA patients and healthy subjects (HS). PC1 and PC2 represent the top Two principal coordinates that captured most of the diversity present. The fraction of diversity captured by the coordinate is given as a percentage and *p* values are determined using the Permanova method. The global composition of bacterial (C) and fungal composition (D) at the phylum level. (E) The α diversity of bacterial microbiota in IUA patients with low and high vaginal pH indicated by the Shannon index. Ns, not significant. Low pH indicates vaginal pH ⩽ 4.6; High pH indicates vaginal pH > 4.6. (F) Principal coordinates analysis of bacterial microbiota in IUA patients with low and high vaginal pH. PC1 and PC2 represent the top Two principal coordinates that captured most of the diversity present. The fraction of diversity captured by the coordinate is given as a percentage and *p* values are determined using the Permanova method. (G) The bacterial composition of IUA patients with low and high vaginal pH at the genus level.

Figure S2. Relative abundance of *Lactobacillus* (A) and *Candida* (B) at the genus level, in the lower FRT (CC and MV) of IUA patients and healthy subjects (HS). The *p* values are determined by Kruskal-Wallis test. Ns, not significant; *, $p < 0.05$.

Figure S3. The protective benefits of *C. parapsilosis* in a rat model of IUA as shown by immunohistochemistry. The expression of Smad2, IL-6, TGF-β1 and Collagen-1 in endometrial and vaginal tissues are shown in the images from each group. In all

panels: Cp, *C. parapsilosis*; Ca, *C. albicans*; Cm, *C. maltosa*.

Figure S4. Altered bacterial composition after treatment with *C. parapsilosis* in a rat model of IUA. The α diversity of bacterial microbiota in each group was estimated by the Chao1 index (A). The diversity of bacteria in each group was estimated by an weighted Unifrac index(B); (C) Bacterial composition at the genus level; (D) Bacterial composition at the phylum level after 14 days; The *p* values are determined by Mann-Whitney test. Ns, not significant; *, $p < 0.05$; **, $p < 0.01$; UC, uterine control group (n=6); UCP, Cp-treated uterine group (n=6); VC, vaginal control group (n=3); VCP, Cp-treated vaginal group (n=6).

Figure S1

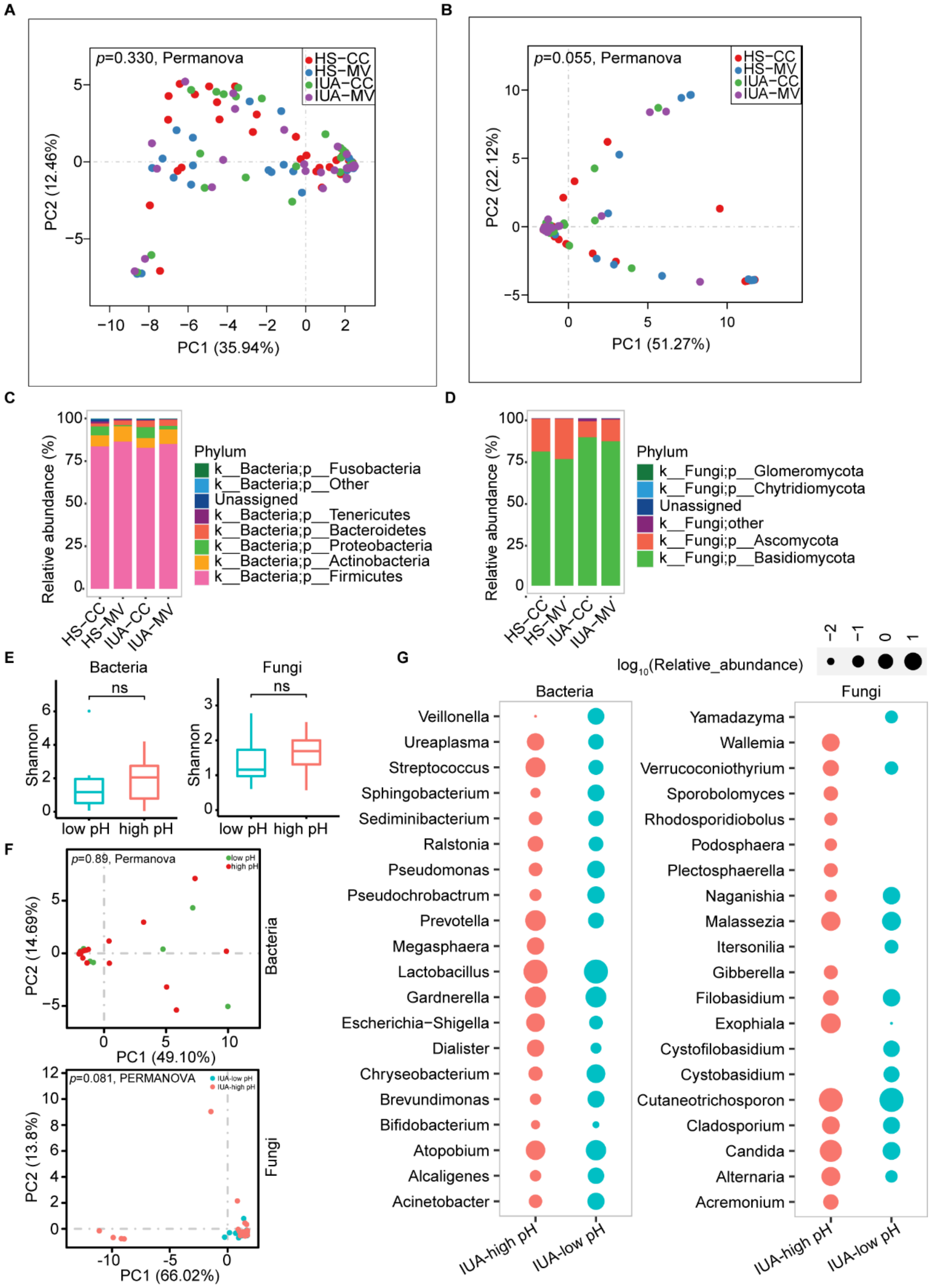

A
p=0.330, Permanova
HS−CC
HS−MV
IUA−CC
IUA−MV
PC2 (12.46%)
PC1 (35.94%)
B
p=0.055, Permanova
HS−CC
HS−MV
IUA−CC
IUA−MV
PC2 (22.12%)
PC1 (51.27%)
C
Relative abundance (%)
Phylum
k__Bacteria;p__Fusobacteria
k__Bacteria;p__Other
Unassigned
k__Bacteria;p__Tenericutes
k__Bacteria;p__Bacteroidetes
k__Bacteria;p__Proteobacteria
k__Bacteria;p__Actinobacteria
k__Bacteria;p__Firmicutes
HS−CC
HS−MV
IUA−CC
IUA−MV
D
Relative abundance (%)
Phylum
k__Fungi;p__Glomeromycota
k__Fungi;p__Chytridiomycota
Unassigned
k__Fungi;other
k__Fungi;p__Ascomycota
k__Fungi;p__Basidiomycota
HS−CC
HS−MV
IUA−CC
IUA−MV
E
Bacteria
ns
Shannon
low pH
high pH
Fungi
ns
Shannon
low pH
high pH
F
p=0.89, Permanova
low pH
high pH
PC2 (14.69%)
PC1 (49.10%)
Bacteria
p=0.081, PERMANOVA
IUA-low pH
IUA-high pH
PC2 (13.8%)
PC1 (66.02%)
Fungi
G
log10(Relative_abundance)
Bacteria
Veillonella
Ureaplasma
Streptococcus
Sphingobacterium
Sediminibacterium
Ralstonia
Pseudomonas
Pseudochrobactrum
Prevotella
Megasphaera
Lactobacillus
Gardnerella
Escherichia−Shigella
Dialister
Chryseobacterium
Brevundimonas
Bifidobacterium
Atopobium
Alcaligenes
Acinetobacter
IUA-high pH
IUA-low pH
Fungi
Yamadazyma
Wallemia
Verrucoconiothyrium
Sporobolomyces
Rhodosporidiobolus
Podosphaera
Plectosphaerella
Naganishia
Malassezia
Itersonilia
Gibberella
Filobasidium
Exophiala
Cystofilobasidium
Cystobasidium
Cutaneotrichosporon
Cladosporium
Candida
Alternaria
Acremonium
IUA-high pH
IUA-low pH

Figure S2

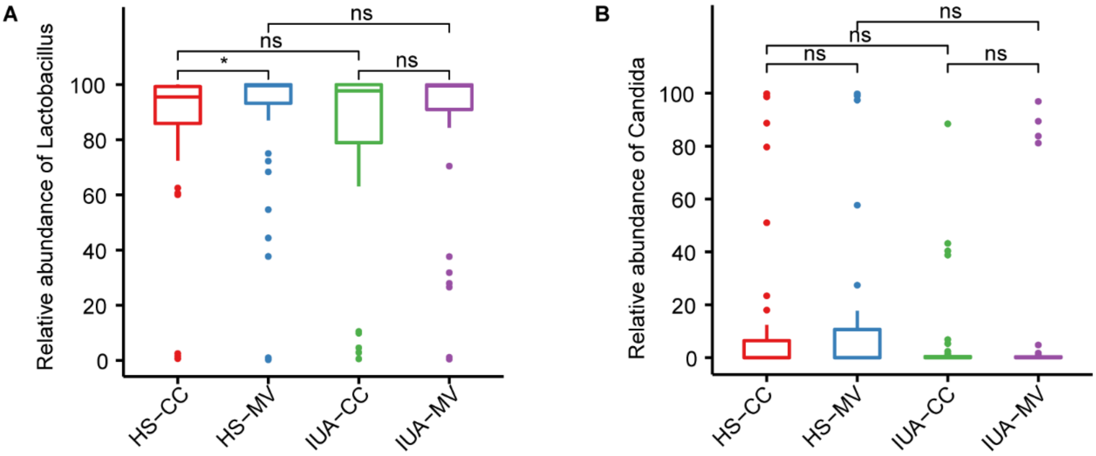

A
Relative abundance of Lactobacillus
0
20
40
60
80
100
ns
ns
*
ns
HS−CC
HS−MV
IUA−CC
IUA−MV
B
Relative abundance of Candida
0
20
40
60
80
100
ns
ns
ns
ns
HS−CC
HS−MV
IUA−CC
IUA−MV

Figure S3

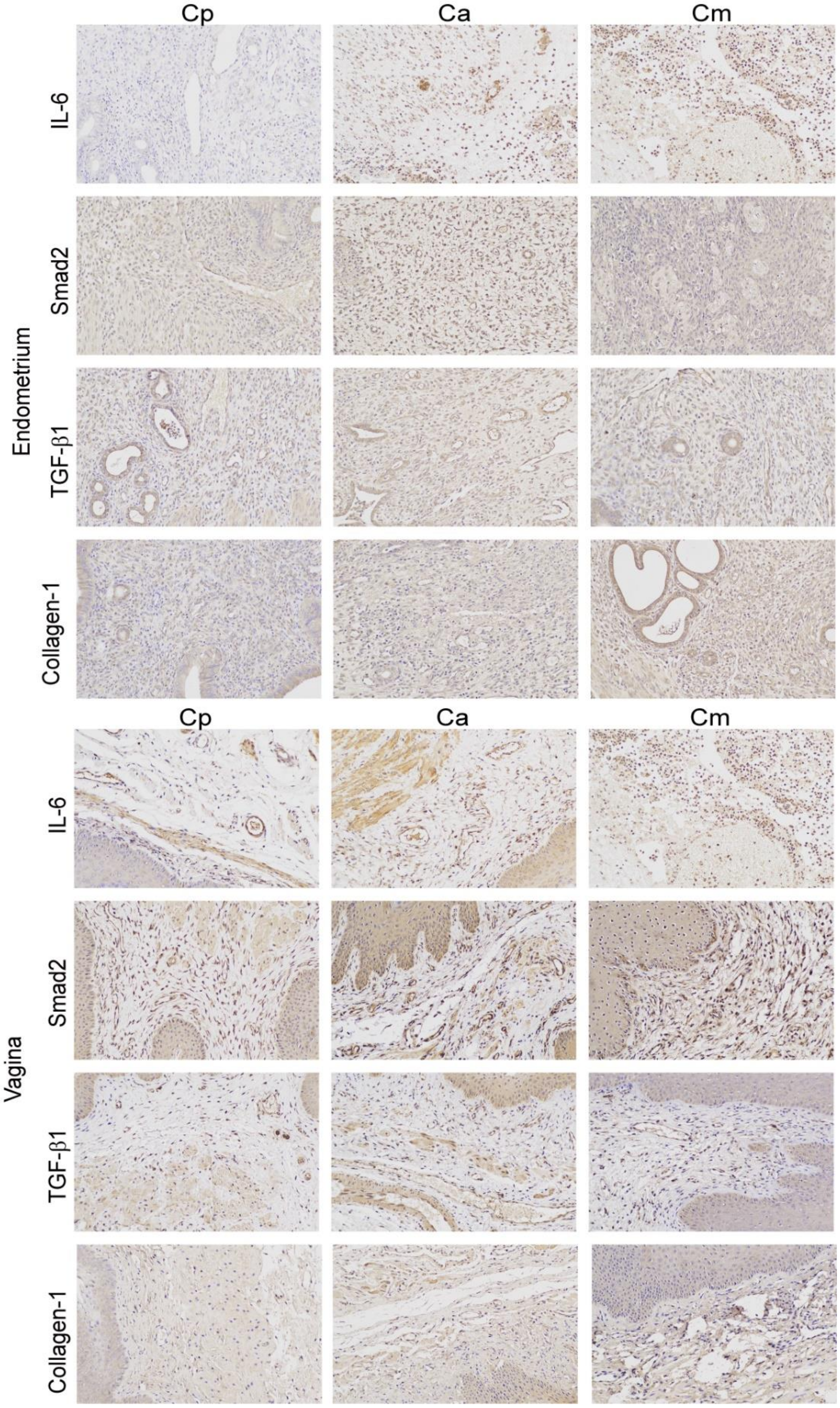
Cp
Ca
Cm
IL-6
Smad2
TGF-β1
Collagen-1
Endometrium
Cp
Ca
Cm
IL-6
Smad2
TGF-β1
Collagen-1
Vagina

Figure S4

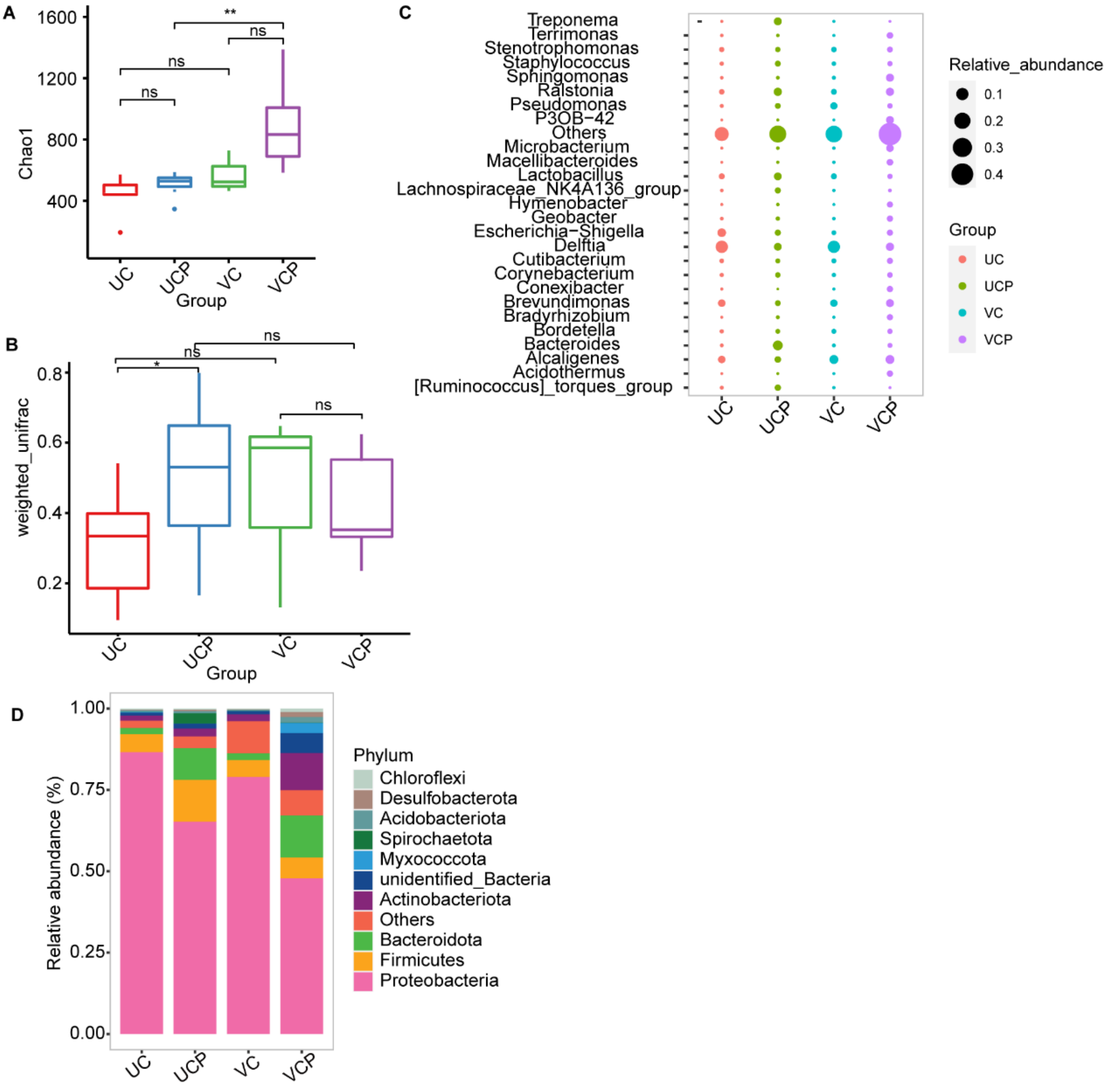
A
Chao1
Group
UC
UCP
VC
VCP
ns
**
B
weighted_unifrac
*
C
Treponema
Terrimonas
Stenotrophomonas
Staphylococcus
Sphingomonas
Ralstonia
Pseudomonas
P3OB−42
Others
Microbacterium
Macellibacteroides
Lactobacillus
Lachnospiraceae_NK4A136_group
Hymenobacter
Geobacter
Escherichia−Shigella
Delftia
Cutibacterium
Corynebacterium
Conexibacter
Brevundimonas
Bradyrhizobium
Bordetella
Bacteroides
Alcaligenes
Acidothermus
[Ruminococcus]_torques_group
Relative_abundance
Group
D
Relative abundance (%)
Phylum
Chloroflexi
Desulfobacterota
Acidobacteriota
Spirochaetota
Myxococcota
unidentified_Bacteria
Actinobacteriota
Others
Bacteroidota
Firmicutes
Proteobacteria
